# A genome-wide association study identifies a novel association between *SDC3* and apparent treatment-resistant hypertension

**DOI:** 10.1186/s12916-022-02665-x

**Published:** 2022-11-30

**Authors:** Xiao Xiao, Rui Li, Cunjin Wu, Yupeng Yan, Mengmeng Yuan, Bing Cui, Yu Zhang, Channa Zhang, Xiaoxia Zhang, Weili Zhang, Rutai Hui, Yibo Wang

**Affiliations:** 1 State Key Laboratory of Cardiovascular Disease, Fuwai Hospital, National Center for Cardiovascular Diseases, Chinese Academy of Medical Sciences and Peking Union Medical College, 167 Beilishi Rd, Beijing, China; 2 Department of Pharmacy, The First Affiliated Hospital, Xi’an Jiaotong University, 277 West Yanta Road, Xi’an, Shaanxi China

**Keywords:** SDC3, aTRH, GWAS, eQTL

## Abstract

**Background:**

Compared with patients who require fewer antihypertensive agents, those with apparent treatment-resistant hypertension (aTRH) are at increased risk for cardiovascular and all-cause mortality, independent of blood pressure control. However, the etiopathogenesis of aTRH is still poorly elucidated.

**Methods:**

We performed a genome-wide association study (GWAS) in first cohort including 586 aTRHs and 871 healthy controls. Next, expression quantitative trait locus (eQTL) analysis was used to identify genes that are regulated by single nucleotide polymorphisms (SNPs) derived from the GWAS. Then, we verified the genes obtained from the eQTL analysis in the validation cohort including 65 aTRHs, 96 hypertensives, and 100 healthy controls through gene expression profiling analysis and real-time quantitative polymerase chain reaction (RT-qPCR) assay.

**Results:**

The GWAS in first cohort revealed four suggestive loci (1p35, 4q13.2-21.1, 5q22-23.2, and 15q11.1-q12) represented by 23 SNPs. The 23 significant SNPs were in or near *LAPTM5*, *SDC3*, *UGT2A1*, *FTMT*, and *NIPA1.* eQTL analysis uncovered 14 SNPs in 1p35 locus all had same regulation directions for *SDC3* and *LAPTM5*. The disease susceptible alleles of SNPs in 1p35 locus were associated with lower gene expression for *SDC3* and higher gene expression for *LAPTM5.* The disease susceptible alleles of SNPs in 4q13.2-21.1 were associated with higher gene expression for *UGT2B4*. GTEx database did not show any statistically significant eQTLs between the SNPs in 5q22-23.2 and 15q11.1-q12 loci and their influenced genes. Then, gene expression profiling analysis in the validation cohort confirmed lower expression of *SDC3* in aTRH but no significant differences on *LAPTM5* and *UGT2B4*, when compared with controls and hypertensives, respectively. RT-qPCR assay further verified the lower expression of *SDC3* in aTRH.

**Conclusions:**

Our study identified a novel association of *SDC3* with aTRH, which contributes to the elucidation of its etiopathogenesis and provides a promising therapeutic target.

**Supplementary Information:**

The online version contains supplementary material available at 10.1186/s12916-022-02665-x.

## Background

Apparent treatment-resistant hypertension (aTRH) is defined as either 2 blood pressures (BPs) of at least 140 mm Hg (systolic) or 90 mm Hg (diastolic) at least 1 month apart during use of 3 antihypertensive agents (including a diuretic) or hypertension requiring 4 antihypertensive classes [1]. The prevalence of aTRH was 19.7% among 10.3 million USA patients with hypertension according to the 2018 American Heart Association Scientific Statement [2]. In China, the prevalence of aTRH in older people (aged ≥ 60–75 years) was 5.97% (169) among 3774 patients with hypertension [3]. Compared with patients who require fewer antihypertensive agents, those with apparent treatment-resistant hypertension are at increased risk for cardiovascular and all-cause mortality, independent of BP control [1]. However, the etiopathogenesis of aTRH is still poorly elucidated. Here, we performed a genome-wide association study (GWAS) to identify genetic polymorphisms associated with aTRH.

## Results

### Discovery of aTRH-associated loci by GWAS

**Fig. 1:**
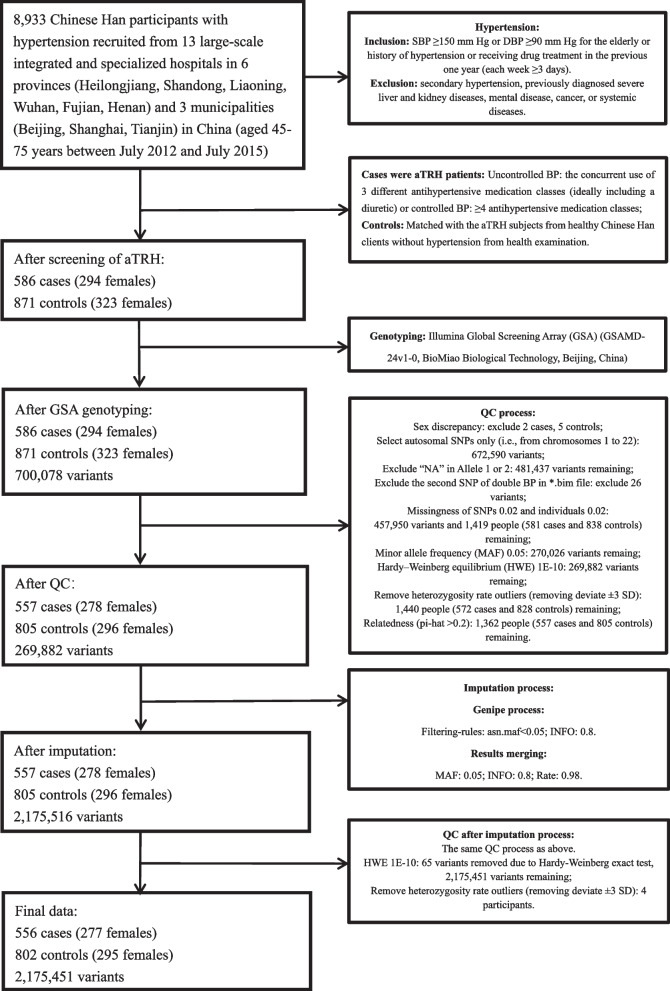
Flow diagram of the screening aTRH patients and the QC results in both genotyping and imputation

**Fig. 2:**
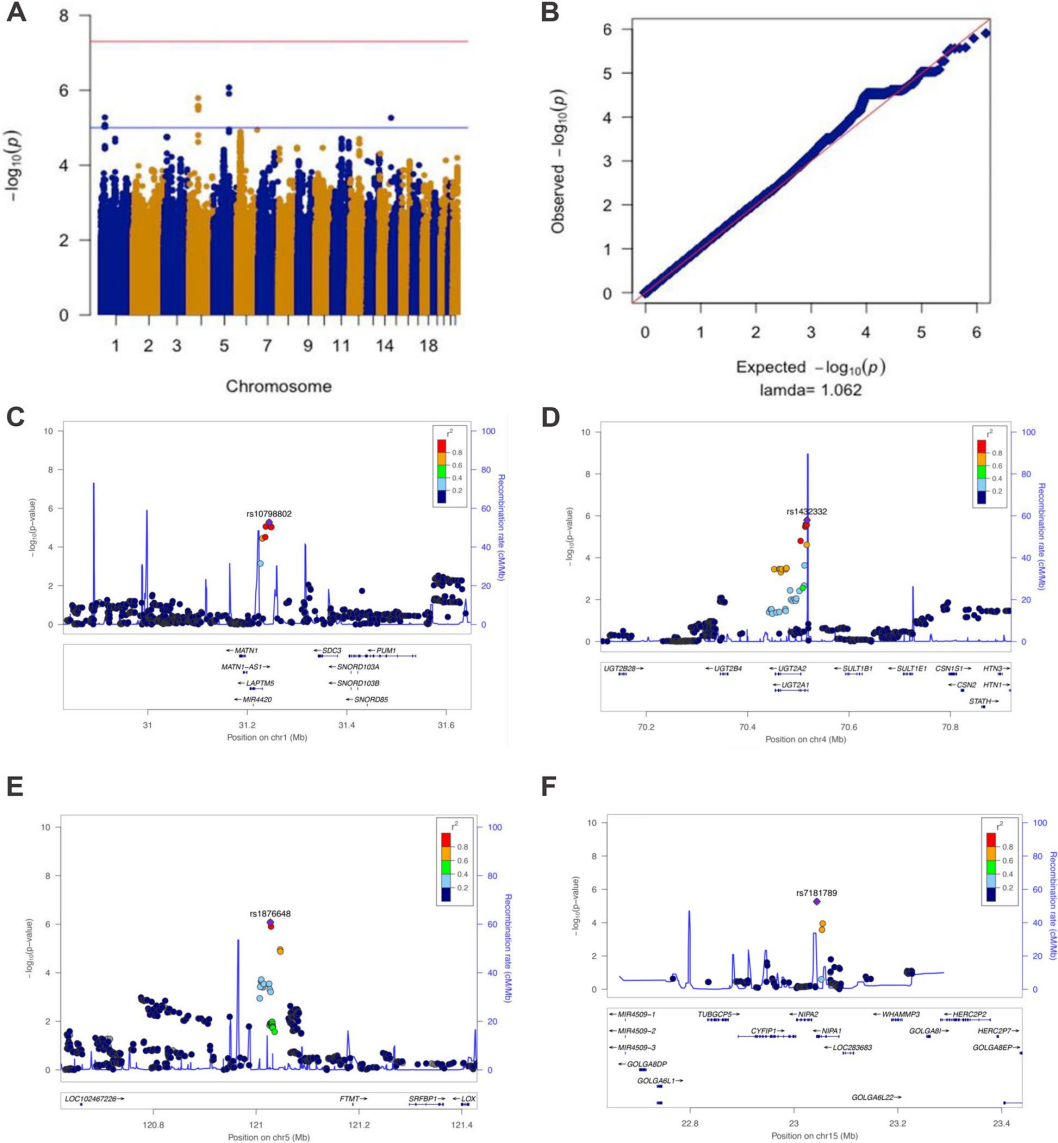
Manhattan and Q-Q plots for aTRH GWAS. Negative log_10_ transformed P-values and physical positions for SNPs in associated regions. All *P* values are negative log_10_ transformed, and both genotyped and imputed SNPs are shown (**A**, **B**). Blue line indicates suggestive association threshold, *P* < 1E−5; red line indicates genome-wide significance threshold, *P* < 5E−8 (**A**). Suggestive associations for aTRH: LocusZoom plots (**C**–**F**) showing the suggestive associated SNPs on chromosome 1 (**C**), 4 (**D**), 5 (**E**), and 15 (**F**) from GWAS of aTRH, respectively. Colors indicate LD between the index SNP (purple) and other SNPs based on HapMap ASN data. The rug plot indicates regional SNP density within 400kb of the suggestive SNPs in the chromosome. The recombination rate overlay (blue line, right x-axis) is based on HapMap ASN data. Gene positions and directions of transcription are annotated based on hg19 /1,000 Genomes Nov 2014 release

Five hundred eighty-six aTRHs and 871 controls were analyzed in the GWAS after quality control (Fig. 1). The Manhattan and Q-Q plots from this aTRH GWAS were shown in Fig. 2 A, B, which included a total of 2,175,451 variants from peripheral blood samples of 1358 individuals (556 aTRHs, 802 healthy controls) (Fig. 1). The genomic inflation factor (lambda) and intercept was 1.062 and 1.0123, respectively (Additional file 1: Table S1), indicating that there was no inflation of *P* values due to the results of population structure or relatedness. Four suggestive loci, represented by 23 SNPs (3 genotyped and 20 imputed) with *P* values between 1e−5 and 1e−7, were listed as follows: 1p35 (rs7542771, rs10798802, rs11299707, rs878465, rs10798803, rs10798804, rs6668307, rs6659862, rs4949297, rs4949179, rs4949298, rs10798805, rs10753235, rs10753236), 4q13.2-21.1 (rs1432330, rs4148279, rs4148277, rs7672805, rs10000435, rs1432332), 5q22-23.2 (rs1876648, rs10056108), and 15q11.1-q12 (rs7181789) (Table 1). The 23 significant SNPs were in or near *LAPTM5*, *SDC3*, *UGT2A1*, *FTMT*, and *NIPA1* (Fig. 2 C–F).

**Table 1:** Suggestive associations (*P* < 1E−5) observed for aTRH

CHR	SNP	Imputed or genotyped	BP	RA	NRA	OR	L95	U95	***P value***	Nearest gene	MAF-case	MAF-control
1p35	rs7542771	Imputed	31237122	T	C	0.588	0.465	0.743	8.76E−06	*LAPTM5-SDC3*	0.150	0.210
1p35	rs10798802	Imputed	31243913	T	C	0.578	0.456	0.732	5.30E−06		0.147	0.209
1p35	rs11299707	Imputed	31246659	AC	A	0.586	0.464	0.742	8.33E−06		0.150	0.211
1p35	rs878465	Imputed	31247089	A	G	0.588	0.465	0.744	9.34E−06		0.150	0.211
1p35	rs10798803	Imputed	31247334	A	C	0.588	0.465	0.744	9.34E−06		0.150	0.211
1p35	rs10798804	Imputed	31247358	T	C	0.588	0.465	0.744	9.34E−06		0.150	0.211
1p35	rs6668307	Imputed	31247514	G	T	0.588	0.465	0.744	9.34E−06		0.150	0.211
1p35	rs6659862	Imputed	31247540	T	C	0.588	0.465	0.744	9.34E−06		0.150	0.211
1p35	rs4949297	Imputed	31247674	C	A	0.588	0.465	0.744	9.34E−06		0.150	0.211
1p35	rs4949179	Imputed	31247811	A	T	0.588	0.465	0.744	9.34E−06		0.150	0.211
1p35	rs4949298	Imputed	31247991	A	G	0.588	0.465	0.744	9.34E−06		0.150	0.211
1p35	rs10798805	Imputed	31248289	G	T	0.588	0.465	0.744	9.34E−06		0.150	0.211
1p35	rs10753235	Imputed	31248432	A	C	0.588	0.465	0.744	9.34E−06		0.150	0.211
1p35	rs10753236	Imputed	31248435	G	T	0.588	0.465	0.744	9.34E−06		0.150	0.211
4q13.2-21.1	rs1432330	Imputed	70513551	G	A	1.553	1.290	1.869	3.33E−06	*UGT2A1*	0.414	0.342
4q13.2-21.1	rs4148279	Genotyped	70514358	C	T	1.563	1.298	1.884	2.61E−06		0.412	0.340
4q13.2-21.1	rs4148277	Imputed	70514829	T	A	1.560	1.295	1.879	2.75E−06		0.413	0.342
4q13.2-21.1	rs7672805	Imputed	70516520	G	A	1.560	1.295	1.879	2.75E−06		0.413	0.342
4q13.2-21.1	rs10000435	Imputed	70516671	C	T	1.563	1.297	1.884	2.71E−06		0.417	0.343
4q13.2-21.1	rs1432332	Imputed	70517108	C	T	1.580	1.311	1.905	1.61E−06		0.416	0.341
5q22-23.2	rs1876648	Genotyped	121027720	A	G	1.581	1.318	1.898	8.38E−07	*LOC102467226-FTMT*	0.475	0.394
5q22-23.2	rs10056108	Imputed	121028770	A	T	1.571	1.309	1.886	1.24E−06		0.476	0.396
15q11.1-q12	rs7181789	Genotyped	23043896	A	G	1.529	1.273	1.837	5.45E−06	*NIPA1*	0.487	0.423

*Chr* chrom genome version of CRCh37/hg19, *Chr* chromosome, *SNP* single-nucleotide polymorphism, *BP* base position, *RA* risk allele, *NRA* non-risk allele, *OR* odds ratio, *L95* lower 95% limit, *U95* upper 95% limit, *MAF* Minor allele frequency

### Expression quantitative trait locus (eQTL) analysis of the 23 SNPs from aTRH GWAS

**Fig. 3:**
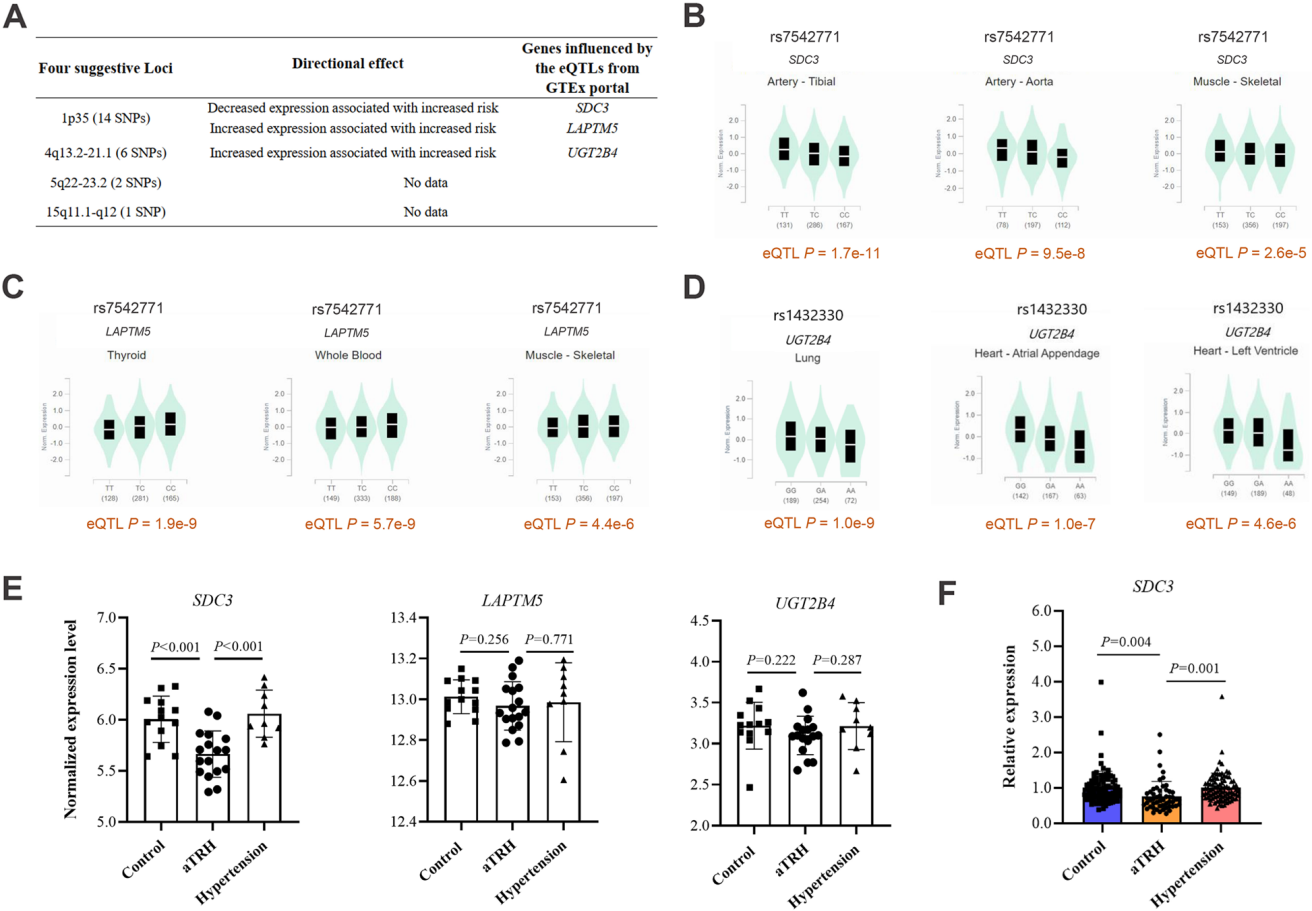
eQTL analysis, gene expression profiling analysis, and RT-qPCR assay identified the low expression of *SDC3* was associated with aTRH. **A** Directional effects of the four suggestive loci from the GTEx portal (https://www.gtexportal.org/home/). **B**, **C** The eQTL plots of the rs7542771 in 1p35 were downloaded from the GTEx portal. Numbers below the genotypes indicate sample size. eQTL *P* values are indicated. **D** The eQTL plots of the rs1432330 in 4q13.2-21.1 were downloaded from the GTEx portal. Numbers below the genotypes indicate sample size. eQTL *P* values are indicated. **E** Peripheral blood gene expression profiling analysis results of *SDC3*, *LAPTM5*, and *UGT2B4* were detected by Affymetrix Human Gene 2.0ST Array in 17 aTRHs, 13 controls, and 9 hypertensives. The error bar indicated 1 SD from the mean. Two tailed *t* test was used for comparison between two groups. **F** The expression difference of *SDC3* was further verified by quantitative polymerase chain reaction experiments in peripheral blood from our previous cohort. The expression of *SDC3* in aTRHs was significantly lower when compared with controls or hypertensives (52 vs 87/87)

We then investigated whether our associated 23 SNPs had been described as eQTLs, using data from the GTEx eQTL database (https://www.gtexportal.org/home/) [4]. We input these SNPs into GTEx eQTL database to identify genes that are regulated by these SNPs (Fig. 3A). For example, rs7542771 in 1p35 locus, eQTL analysis showed that TC/CC genotype had a significantly lower *SDC3* expression than TT genotype (Fig. 3B, Additional file 1: Figure S1). The frequency of allele C was 0.79 in the controls and 0.85 in the aTRHs (Table 1), so a lower expression of *SDC3* was speculated to be associated with aTRHs. 14 SNPs in 1p35 locus all had same regulation directions for *SDC3* and *LAPTM5* in eQTLs analysis. The disease susceptible alleles of SNPs in 1p35 locus were associated with lower gene expressions for *SDC3* in both artery and skeletal tissues and associated with higher gene expressions for *LAPTM5* in thyroid, whole blood, and skeletal tissues in the GTEx database (Fig. 3C, Additional file 1: Figure S2). Six SNPs in 4q13.2-21.1 locus all had same regulation directions for *UGT2B4*. The disease susceptible alleles of SNPs in 4q13.2-21.1 were associated with higher gene expressions for *UGT2B4* in both lung and heart tissues (Fig. 3D, Additional file 1: Figure S3). Besides, GTEx database did not show any statistically significant eQTLs between the SNPs in 5q22-23.2 and 15q11.1-q12 loci and their influenced genes in any of the tissues present in the database.

### Validation of the eQTL results in another cohort

The baseline demographics of the validation cohort was displayed in Table 2. We randomly selected 17 aTRHs, 9 hypertensives and 13 controls for expression profile chip analysis. The results showed lower expression of *SDC3* in the peripheral blood of the aTRH group compared with control group (unpaired *t* test, *P* < 0.001) and hypertension group (unpaired *t* test, *P* < 0.001), respectively. However, no significant differences on the expression of either *LAPTM5* or *UGT2B4* were observed between aTRH group and control group or hypertension group. (Fig. 3E).

**Table 2:** Baseline characteristics of study participants

Characteristics	GWAS cohort			Validation cohort			
	aTRHs	Controls	*P* value	aTRHs	Hypertensives	Controls	*P* value
Number of samples	586	871		65	96	100	
Age (year)	58.4 ± 11.6	58.7 ± 9.7	0.593	59.3 ± 5.9	60.6 ± 6.7	59.8 ± 6.6	0.434
Sex, male (%)	292 (49.8)	548 (62.9)	< 0.001	32 (49.2)	48 (50.0)	51 (51.0)	0.987
Body mass index (kg/m^2^)	26.5 ± 3.6	26.2 ± 3.9	0.138	23.1 ± 1.9	22.6 ± 1.5	22.1 ± 1.4	< 0.001
Waist circumference (cm)	91.6 ± 8.7	83.7 ± 5.6	< 0.001	90.5 ± 8.4	84.7 ± 6.3	83.2 ± 5.8	< 0.001
SBP (mm Hg)	159.8 ± 24.8	117.7 ± 12.1	< 0.001	158.2 ± 19.1	144.3 ± 14.9	111.4 ± 7.6	< 0.001
DBP (mm Hg)	92.0 ± 15.4	74.3 ± 8.9	< 0.001	94.9 ± 11.3	87.3 ± 10.5	69.2 ± 5.3	< 0.001
Smoke (%)	124 (21.2)	164 (18.8)	0.273	14 (21.5)	19 (19.8)	19 (19.0)	0.912
Alcohol (%)	91 (15.6)	141 (16.2)	0.736	11 (16.9)	16 (16.7)	16 (16.0)	0.980
Hyperlipidemia (%)	319 (54.4)	0	< 0.001	36 (55.4)	0	0	< 0.001
Coronary heart disease (%)	171 (29.2)	0	< 0.001	19 (29.2)	2 (2.1)	0	< 0.001
Type 2 diabetes mellitus (%)	213 (36.3)	0	< 0.001	25 (38.5)	2 (2.1)	0	< 0.001
Stroke (%)	149 (25.4)	0	< 0.001	14 (21.5)	0	0	< 0.001
Family history of hypertension (%)	379 (64.7)	0	< 0.001	41 (63.1)	59 (61.5)	0	< 0.001
Antihypertensive drugs							
Calcium channel blocker, *n* (%)	530 (90.4)	0	< 0.001	59 (90.8)	63 (65.6)	0	< 0.001
ARB or ACEi, *n* (%)	372 (63.5)	0	< 0.001	43 (66.2)	46 (47.9)	0	< 0.001
Beta-blocker, *n* (%)	295 (50.3)	0	< 0.001	34 (52.3)	33 (34.4)	0	< 0.001
Diuretics, *n* (%)	511 (87.2)	0	< 0.001	57 (87.7)	10 (10.4)	0	< 0.001

*aTRH* apparent treatment resistant hypertension, *SBP* systolic blood pressure, *DBP* diastolic blood pressure; smoking and drinking status counts only current smokers and drinkers, excluding those who have quit, *ARB* angiotensin receptor blocker, *ACEi* angiotensin-converting enzyme inhibitor; data were given as mean ± SD. Differences in continuous variables between two groups were compared with a unpaired t-test and differences in categorical variables were measured with a chi-square test. Two-tailed *P* value of < 0.05 was considered statistically significant

Therefore, *SDC3* was the most prominent gene related to aTRH. We also randomly selected 6 SNPs related to *SDC3* in 1p35 locus to detect their frequency in cases and controls using MassARRAY high-throughput DNA analysis to confirm the results of GWAS, which was consistent (Additional file 1: Table S2). Due to the small sample size of the expression profiling data, we further determined *SDC3* expression in the rest samples of the validation cohort (52 aTRHs, 87 hypertensives, and 87 controls) using real-time quantitative polymerase chain reaction (RT-qPCR) assay. RT-qPCR assay showed the *SDC3* expression levels in the aTRHs were lower than those in the controls (unpaired *t* test, *P* = 0.004) and the hypertensives (unpaired *t* test, *P* = 0.001) (Fig. 3F), respectively.

## Methods

The data that support the findings of this study are available from the corresponding author upon reasonable request.

### Study population

The GWAS aTRH patients were screened from a cohort of 8933 Chinese Han participants with hypertension aged 45–75 years between July 2012 and July 201 5[3], who were recruited from 13 large-scale integrated and specialized hospitals in 6 provinces (Heilongjiang, Shandong, Liaoning, Wuhan, Fujian, Henan) and 3 municipalities (Beijing, Shanghai, Tianjin) in China. The inclusion and exclusion criteria of hypertension were as follows: the definition of hypertension is SBP ≥ 150 mmHg or DBP ≥ 90 mmHg for the history of hypertension or taking medicine treatment during the previous 1-year (≥ 3 days in each week) patients or for the elderly. If the participants had secondary hypertension, they would be excluded. Sleep apnea was also ruled out due to its frequent coexists with hypertension. White coat hypertension patients were not excluded through measuring ambulatory monitoring BP. Besides, all participants were not diagnosed systemic diseases, severe liver and kidney diseases, cancers, or mental diseases previously. aTRH was defined as BP above goal though using 3 different antihypertensive agent classes, ideally including a diuretic or achieving BP control through ≥ 4 antihypertensive drug classes. The pseudo resistance issues were not ruled out as potential factor for the ongoing hypertension. At the beginning of the enrollment, controls were matched with the suspected aTRHs for age, sex, and region of residence from healthy Chinese Han clients without hypertension from health examination, and then some cases were excluded due to controlled hypertension. Finally, 586 aTRHs and 871 controls were analyzed in the GWAS.

The validation cohort included 65 aTRHs, 96 hypertensives, and 100 controls, which was screened from a previous cohort from Rizhao City, in the northern region of China from 2009 to 2010 which has been described previously [5, 6]. The following strict inclusion criteria were used for both hypertension patients and controls: (1) Chinese Han people, (2) aged from 50–77 years, (3) BMI ranged from 19.4 kg/m^2^ to 24.6 kg/m^2^, and (4) subjects were excluded when they had any known diseases including thyroid disease, hematological diseases, heart failure, valvular heart disease, liver or kidney dysfunctions, infections, autoimmune diseases, or tumors. The controls had normal blood pressure (SBP ≤ 120 mmHg and DBP ≤ 80 mmHg) and no history of hypertension. On the other side, the patients with essential hypertension had (1) elevated blood pressure SBP ≥ 160 mmHg and DBP ≥ 100 mmHg (antihypertensive drug treatment in a patient with a history of hypertension SBP ≥ 160 mmHg and DBP ≥ 100 mmHg is an alternate indicator) and (2) hypertension history for more than 4 years. aTRH was defined as same as above.

The study was approved by the Fuwai Hospital ethics committee, Peking Union Medical College, and conducted based on the Declaration of Helsinki. Written informed consents were obtained from all subjects. The baseline demographics of the two cohort were displayed in Table 2.

### BP measurement in the two cohorts

BP was measured by trained professionals with a validated oscillometric BP monitor (Omron HEM-907XL) with appropriately sized arm cuffs in the subject’s right arm and a standardized protocol. Patients were instructed to sit quietly for 5 min before taking the measurement. All participants were asked to avoid alcohol, smoking, coffee/tea, and exercise for at least 30 min before the BP measurement. BP was taken three times: 30 s apart and after 5 min of rest, and an average of three readings was used as analyzed BP levels [7].

### Genotyping, quality control and imputation of the GWAS data

The genomic DNA was extracted from peripheral blood of all the participants. Genotyping in all individuals was done using Illumina Global Screening Array (GSA) (GSAMD-24v1-0, BioMiao Biological Technology, Beijing, China). The quality control measure for all samples was applied by using PLINK 1. 9[8] (www.cog-genomics.org/plink/1.9/) and language R (https://www.r-project.org/). Briefly, the exclusion criteria were as follows: individuals and markers ≤ 98% genotype calls, minor allele frequency ≤ 5%, and Hardy-Weinberg equilibrium (*P* < 1E−10). Sex was matched with genetic data. Autosomal SNPs only (i.e., from chromosomes 1 to 22) were selected. Outliers of subjects were removed by a criterion of deviate ± 3 SD from heterozygosity rate mean of the samples. The cryptic relatedness was assessed and related pairs (defined as pairs with pi-hat > 0.2) were removed [9]. The ethnicity of the samples was ascertained by carrying out a multi-dimensional scaling (MDS) analysis of the genotypes, and the cluster of all individuals was concentrated in the Asian (ASN) descent (Additional file 2). A total of 269,882 variants of 1362 samples were directly genotyped after quality control and were available for the following imputation. The whole-genome imputation was conducted by utilizing the GENome-wide Imputation PipelinE (genipe) module [10], which provides an easy and efficient pattern for performing genome-wide imputation analysis through using three commonly utilized tools PLINK, SHAPEIT, and IMPUTE2. PLINK was applied for the initial genetics data management; SHAPEIT was implemented for the loci strand verification and phasing step; IMPUTE2 was carried out in 5-Mb segments for the imputation process. Pipeline of the genipe package was used for managing the intermediate files produced by the tools at different stages. The 1000 Genomes Phase I integrated haplotypes in NCBI build 37 (hg19) coordinated data [11] of ASN ancestry was used as a reference, which included Japanese individuals from Tokyo, Japan (89), and Han Chinese individuals from South (100) and Beijing, China (97). A high genotype information value (info > 0.8) was applied for imputing single-nucleotide polymorphisms (SNPs) process. The imputed and genotyped processes were utilized the same quality control criteria, yielding a total of 2,175,451 variants from 1358 individuals (786 males, 572 females; 556 cases, 802 controls).

### Data analysis of the GWAS data

To detect the genetic association between the SNPs and aTRH, a GWAS logistic regression model was performed in PLINK 1.9 [8] by adjusting for sex and the top 10 components from the MDS analysis as covariates. The analyses were performed with both genotyped and imputed SNP data. The significance threshold was based on the Bonferroni correction for multiple tests. Any SNPs with *P* < 5E−8 were considered to be genome-wide significant. Any SNPs with 5E−8 < *P* < 1E−5 were considered as suggestive significance SNPs associated with aTRH. The SnpEff v3.6 [12] was applied for making a variant annotation for the SNPs with suggestive significance. R statistical package (R Foundation for Statistical Computing) was implemented for calculating genomic inflation factors (lambda), generating Manhattan and quantile-quantile (Q-Q) plots. LocusZoom [13, 14] (http://csg.sph.umich.edu/locuszoom/) was carried out to visualize regions around SNPs with suggestive significance. Linkage disequilibrium (LD) scores were calculated by LDSC [15, 16]. The general information of aTRH GWAS was given in Additional file 1: Table S1.

### Characterization of genomic risk loci based on aTRH GWAS

Genomic risk loci for aTRH susceptibility were defined based on aTRH GWAS summary statistics, and several subsets of SNPs in the loci were named by the following criteria: (i) independently significant SNPs: *P* < 1E−5 and independent from each other at r2 < 0.6; (ii) candidate SNPs: r2 ≥ 0.6 with one of the independent significant SNPs, all of those candidate SNPs including non-GWAS-tagged SNPs will be submitted to further gene mapping; (iii) independent lead SNPs: independent significant SNPs and independent from each other at r2 < 0.1; (iv) genomic risk loci: merging lead SNPs within a 250 kb window, containing multiple independent significant SNPs and/or lead SNPs.

### eQTL analysis using the GTEx database

To investigate if any of the variants are eQTLs, we used the GTEx portal web tool of the GTEx database (GTEx Analysis Release V8 (dbGaP Accession phs000424.v8.p2)) with default parameters to obtain the data. Our eQTL analysis was carried out by logging onto https://gtexportal.org, typing in the corresponding rs number of identified variants, and checking if they have any associated eQTLs.

### Expression profile chip analysis

The Affymetrix human gene 2.0 ST profile chip was used to detect the whole transcript level. About 2.5 mL of venous blood was collected with PAXgene (Qiagen) venous blood collection tube, blood was used to extract RNA for further research [6].

### Real-time quantitative polymerase chain reaction

Total RNAs from tissues or cell lines were isolated using TRIzol and subjected to reverse transcription with according to the manufacturer’s instructions. For quantitative PCR, cDNA fragments were subjected to SYBR Green RT-PCR (11203ES03, YEASEN, China) using ABI system. Analysis of *SDC3* mRNA expression was performed using the primers as follows: 5′-TGGCGCAGTGAGAACTTCG-3′ (forward) and 5′-CCCCGAGTAGAGGTCATCCAG-3′ (reverse). Specific PCRs (20 μL) were set up as described previously [17]. The quantitative measures were obtained using the 2^ΔΔCT^ method.

### Statistical analysis

Data are reported as mean ± standard deviation (SD) for continuous variables and as frequency for categorical variables. Differences in continuous variables between two groups were compared with an unpaired *t*-test and differences in categorical variables were measured with a chi-square test. Two-tailed *P* value of < 0.05 was considered statistically significant. Differences between multiple groups were performed by analysis of variance (ANOVA). All statistical analysis involved using SPSS Statistics 17.0.

## Discussion

This study represents the first GWAS of aTRH in the Chinese Han population. Over the past few years, GWAS and exome sequencing studies have resulted in an unparalleled burst of discovery in the genetics of blood pressure regulation and hypertension [18–21]. More importantly, GWAS, while expanding the list of common genetic variants associated with blood pressure and hypertension, are also uncovering novel pathways of blood pressure regulation that augur a new era of novel drug development, repurposing, and stratification in the management of hypertension [18]. Although the genetics of blood pressure and essential hypertension have been extensively investigated, the evidence focused on the genetic studies of aTRH is still limited, and the procurable genetic data about resistant hypertension (RH) are hindered by power and scope [22, 23]. Recently, a few GWASs have identified some possibly significant SNPs for susceptibility to RH/aTRH in the American, European, African, and Japanese population [22, 24–28]. Our research fills the gap in the current state of knowledge of genetic polymorphisms associated with aTRH in Asian Chinese population and provides a basis for future studies.

Here, we performed a GWAS of aTRH in Asian Chinese population, which was from a large-scale screening of hypertension data (8,933 hypertensive patients) [3]. In this study, we found 4 suggestive loci, represented by 23 SNPs. GTEx and the eQTL catalogue provided us with the unique opportunity to investigate the associations among genotype and gene expression [29]. Through eQTL analysis, we associated these aTRH related SNPs with the genes they could potentially regulate [30]. Three candidate genes: *SDC3*, *LAPTM5*, and *UGT2B4* were identified that were regulated by SNPs in 1p53 and 4q13.2-21.1 locus.

Lysosomal-associated transmembrane protein 5 (LAPTM5) [31], involved in lysosome biogenesis and function, has been identified as a potential blood biomarker for hypertensive patients with left ventricular hypertrophy [32] and was upregulated in insulin resistance and obesity [33, 34]. Uridine 5′-diphosphate-glucuronosyltransferase 2B4 (UGT2B4), reported linked to metabolism, could convert hydrophobic bile acids into more hydrophilic glucuronide derivatives [35]. Higher level of UGT2B4 was observed in at least four of five primary human hepatocellular carcinogenesis patients compared to matched nontumorous liver tissues [36]. Syndecan-3 (SDC3), a heparin sulfate proteoglycan, had been found by previous studies to be linked with energy balance and obesity [37]. Then, gene expression profiling analysis in the validation cohort showed lower expression of *SDC3* in aTRH but no significant differences on the expression of either *LAPTM5* or *UGT2B4* were observed. We further confirmed lower expression of *SDC3* in aTRH using RT-qPCR assay.

It has been reported that food deprivation increased hypothalamic syndecan-3 levels more than 4-fold above that of ad libitum fed mice and the elevated levels of syndecan-3 fall with refeeding [38]. The data of mouse model uncovered that *SDC3* null mice improved lipid metabolism [39], but our study identified the low expression of *SDC3* was associated with aTRH. These results suggested that while closely related, aTRH and obesity were regulated by SDC3 via different mechanisms.

GWAS on hypertension have contributed to the depth of understanding of the genetics origins of hypertension [40]. The genetic architecture of blood pressure encompasses approximately 30 genes, with rare variants involved in blood pressure dysregulation and > 1477 common SNPs associated with blood pressure [19]. But our results do not correlate with previous hypertension researches. The reasons for this may be hypertension is mainly related to blood pressure regulation [20, 41–44], while resistant hypertension is closely related to metabolism [45–49], like fatty acid, lipid, amino acid, and purine metabolism. So, it is not surprising that there are no repeated results between our study and theirs.

Recently, a few GWASs have identified some possibly significant SNPs for susceptibility to RH/aTRH in the American, European, African, and Japanese population [22, 24–28], such as ATPase, Ca++ transporting, plasma membrane 1 (*ATP2B1*) rs12817819 [28], DLG-associated protein 1 (*DLGAP1*) rs1442386 [27], cytokine-dependent hematopoietic cell linker (*CLNK*) rs13144136 [25], castor zinc finger 1 (*CASZ1*) rs12046278 [22], and protein tyrosine phosphatase receptor type D (*PTPRD*) rs324498 [24]. In these studies including ours, only *PTPRD* rs324498 was reported in three cohorts, while no more SNPs were reported in two or more cohorts [22, 24–28]. It could be due to lots of reasons. The first is from different genetic background. The second is the inclusion criteria difference. The third is from the control cohort. Normal healthy population was used in some studies, while mild hypertension or controllable hypertension population was used in other studies. Metabolism of antihypertensive agents plays an important role in aTRH. So environmental factors such as diet style, exercise, and stress were also involved. It could be due to the same inclusion criteria and similar genetic background that we confirmed our results in the validation cohort in our study.

The study has some limitations. First, GSA BeadChip was used for genotyping in this GWAS, which is an effective chip for the genetic risk screening in the large-scale populations globally, containing a total of 642,824 markers and performing perfect filling performance across 26 continental populations. Therefore, the specificity of the GSA SNP genotyping results for Chinese population is not as good as Asian Screening. Secondly, the samples of the GWAS cohort are relatively small; however, they are carefully and strictly screening from a large-scale screening of hypertension data (8933 hypertensive patients) with strict inclusion and exclusion criteria. Finally, this study was the only one multi-center study of Chinese Han population with aTRH in China, which should be further verified in non-Han populations.

## Conclusions

In conclusion, the GWAS in the first cohort suggested four suggestive loci, represented by 23 SNPs associated with aTRH. Next, eQTL analysis uncovered *SDC3* and *LAPTM5* were regulated by the SNPs in 1p35 locus, and *UGT2B4* was regulated by the SNPs in 4q13.2-21.1 locus. Then, gene expression profiling analysis and RT-qPCR assay in the validation cohort showed that low expression of *SDC3* was observed in aTRH. Our study identified a novel association of *SDC3* with aTRH, which contributes to the elucidation of its etiopathogenesis and provides a promising therapeutic target.

## Supplementary Information

**Additional file 1: Table S1.** The general information of aTRH GWAS. **Table S2.** MassARRAY high-throughput DNA analysis of six randomly selected SNPs related to *SDC3*. **Figure S1.** Multi-tissue eQTL comparisons of rs7542771 in 1p35 locus for *SDC3* by GTEx database. **Figure S2.** Multi-tissue eQTL comparisons of rs7542771 in 1p35 locus for *LAPTM5* by GTEx database. **Figure S3.** Multi-tissue eQTL comparisons of rs1432330 in 4q13.2-21.1 locus for *UGT2B4* by GTEx database.

**Additional file 2.** Multi-dimensional scaling (MDS) analysis of the genotypes.

## Data Availability

Restrictions apply to the availability of all data analyzed during this study to preserve patient confidentiality or because they were used under license. The corresponding author will, on request, detail the restrictions and any conditions under which access to some data may be provided.
