# Correction: A genome-wide association study identifies a novel association between SDC3 and apparent treatment-resistant hypertension

**DOI:** 10.1186/s12916-022-02717-2

**Published:** 2022-12-20

**Authors:** Xiao Xiao, Rui Li, Cunjin Wu, Yupeng Yan, Mengmeng Yuan, Bing Cui, Yu Zhang, Channa Zhang, Xiaoxia Zhang, Weili Zhang, Rutai Hui, Yibo Wang

**Affiliations:** 1 State Key Laboratory of Cardiovascular Disease, Fuwai Hospital, National Center for Cardiovascular Diseases, Chinese Academy of Medical Sciences and Peking Union Medical College, 167 Beilishi Rd, Beijing, China; 2 Department of Pharmacy, The First Affiliated Hospital, Xi’an Jiaotong University, 277 West Yanta Road, Xi’an, Shaanxi China

**Correction: BMC Med 20, 463 (2022)**

**https://doi.org/10.1186/s12916-022-02665-x**

The original article [1] omitted equal contribution assignment to Xiao Xiao and Rui Li due to a mistake by the typesetting team. The attribution has been restored in the original article.
