# Supplementary material for: A genome-wide association study identifies a novel association between SDC3 and apparent treatment-resistant hypertension
Source: BMC Med. 2022 Nov 30;20:463. doi: 10.1186/s12916-022-02665-x (PMC9710180; doi:10.1186/s12916-022-02665-x)
Supplement: Supplementary file 1 — Additional file 1: Table S1. The general information of aTRH GWAS. Table S2. MassARRAY high-throughput DNA analysis of six randomly selected SNPs related to SDC3. Figure S1. Multi-tissue eQTL comparisons of rs7542771 in 1p35 locus for SDC3 by GTEx database. Figure S2. Multi-tissue eQTL comparisons of rs7542771 in 1p35 locus for LAPTM5 by GTEx database. Figure S3. Multi-tissue eQTL comparisons of rs1432330 in 4q13.2-21.1 locus for UGT2B4 by GTEx database. [file 12916_2022_2665_MOESM1_ESM.docx]

| **Table S1. The general information of aTRH GWAS.** | |
| --- | --- |
| **Feature** | **Values** |
| Ancestry population | Asian (Chinese) |
| Case | 556 |
| Control | 802 |
| Number of SNPs | 2175451 |
| Lambda | 1.062 |
| h2 | 0.5245 (0.2184) |
| LambdaGC (s.e.) | 1.0588 |
| Mean χ2 | 1.047 |
| Intercept (s.e.) | 1.0123 (0.0071) |
| LambdaGC, estimated LambdaGC; χ2, estimated chi square; Intercept, estimated single trait intercept. h2, LambdaGC, Mean χ2, and Intercept were estimated by the LD score regression [81] based on GWAS summary data of aTRH.  **Table S2. MassARRAY high-throughput DNA analysis of six randomly selected SNPs related to *SDC3*.** | |

We validated six randomly selected SNPs related to SDC3 using MassARRAY high-throughput DNA analysis. The Genotyping was further performed using MassARRAY high-throughput DNA analysis^9^ with matrix-assisted laser desorption/ionization time-of-flight mass spectrometry (Sequenom Inc, San Diego, CA). The primers were designed by MassARRAY Assay Design software (version 3.1). Variants were genotyped using iPLEX Gold technology (Sequenom) followed by an automated data analysis with the TYPER RT software version 4.0. The results were shown as below.

| CHR | SNP | Genotype-case (586) | | | Genotype-control (871) | | | Minor allele | MAF-case | MAF-control |
| --- | --- | --- | --- | --- | --- | --- | --- | --- | --- | --- |
| 1p35 | rs7542771 | CC(423) | CT(150) | TT(13) | CC(565) | CT(245) | TT(61) | T | 0.150 | 0.211 |
| 1p35 | rs10798802 | CC(423) | CT(150) | TT(13) | CC(564) | CT(246) | TT(61) | T | 0.150 | 0.211 |
| 1p35 | rs6668307 | TT(421) | TG(152) | GG(13) | TT(565) | TG(245) | GG(61) | G | 0.152 | 0.211 |
| 1p35 | rs6659862 | CC(423) | CT(150) | TT(13) | CC(565) | CT(245) | TT(61) | T | 0.150 | 0.211 |
| 1p35 | rs10798805 | TT(423) | TG(150) | GG(13) | TT(565) | TG(245) | GG(61) | G | 0.150 | 0.211 |

Chr, chrom genome version of CRCh37/hg19; single-nucleotide polymorphism; MAF, Minor allele frequency.

**Figure S1. Multi-tissue eQTL comparisons of rs7542771 in 1p35 locus for *SDC3* by GTEx database**


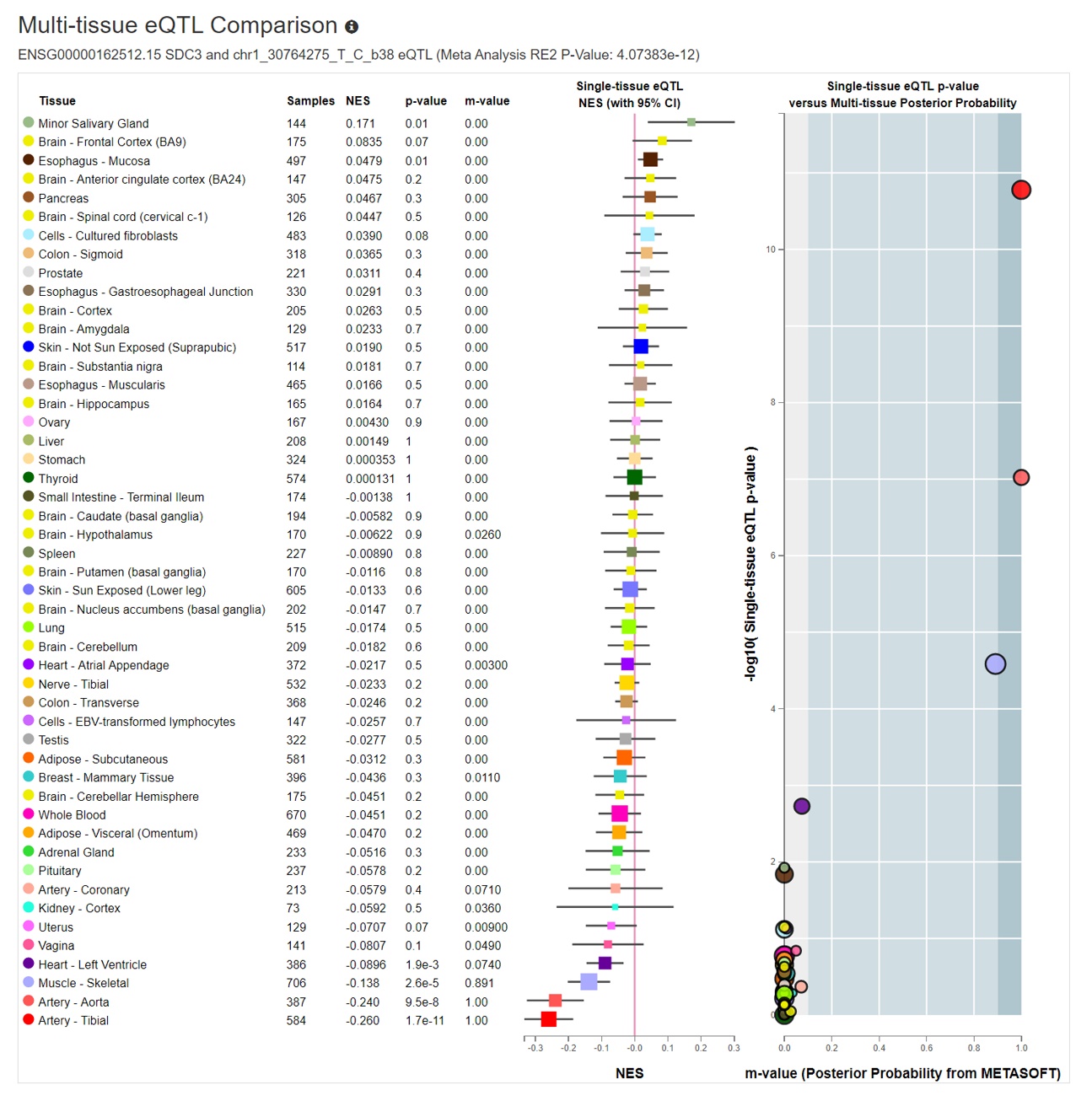


**Figure S2. Multi-tissue eQTL comparisons of rs7542771 in 1p35 locus for *LAPTM5* by GTEx database**


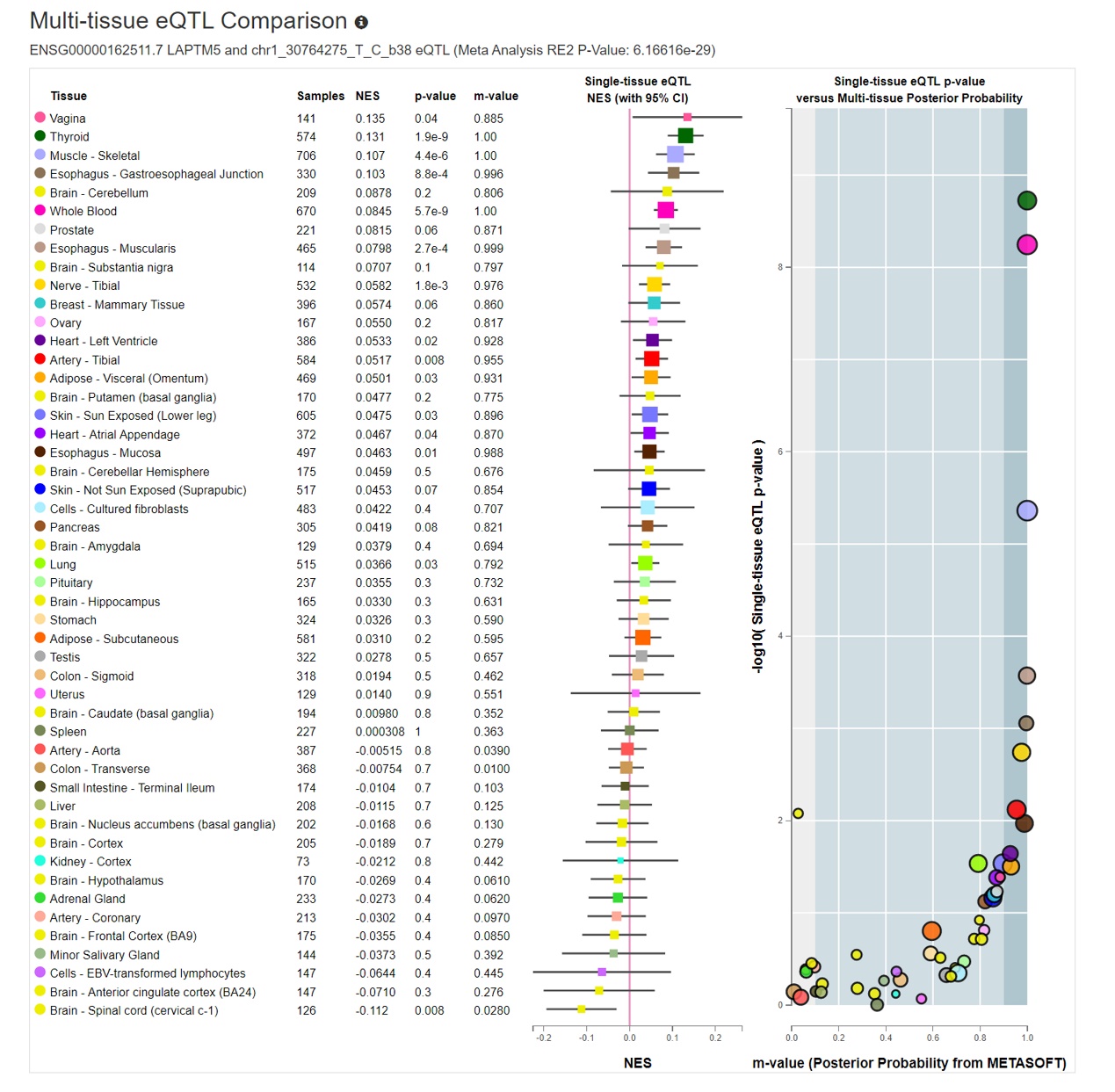


**Figure S3. Multi-tissue eQTL comparisons of rs1432330 in 4q13.2-21.1 locus for *UGT2B4* by GTEx database**

**
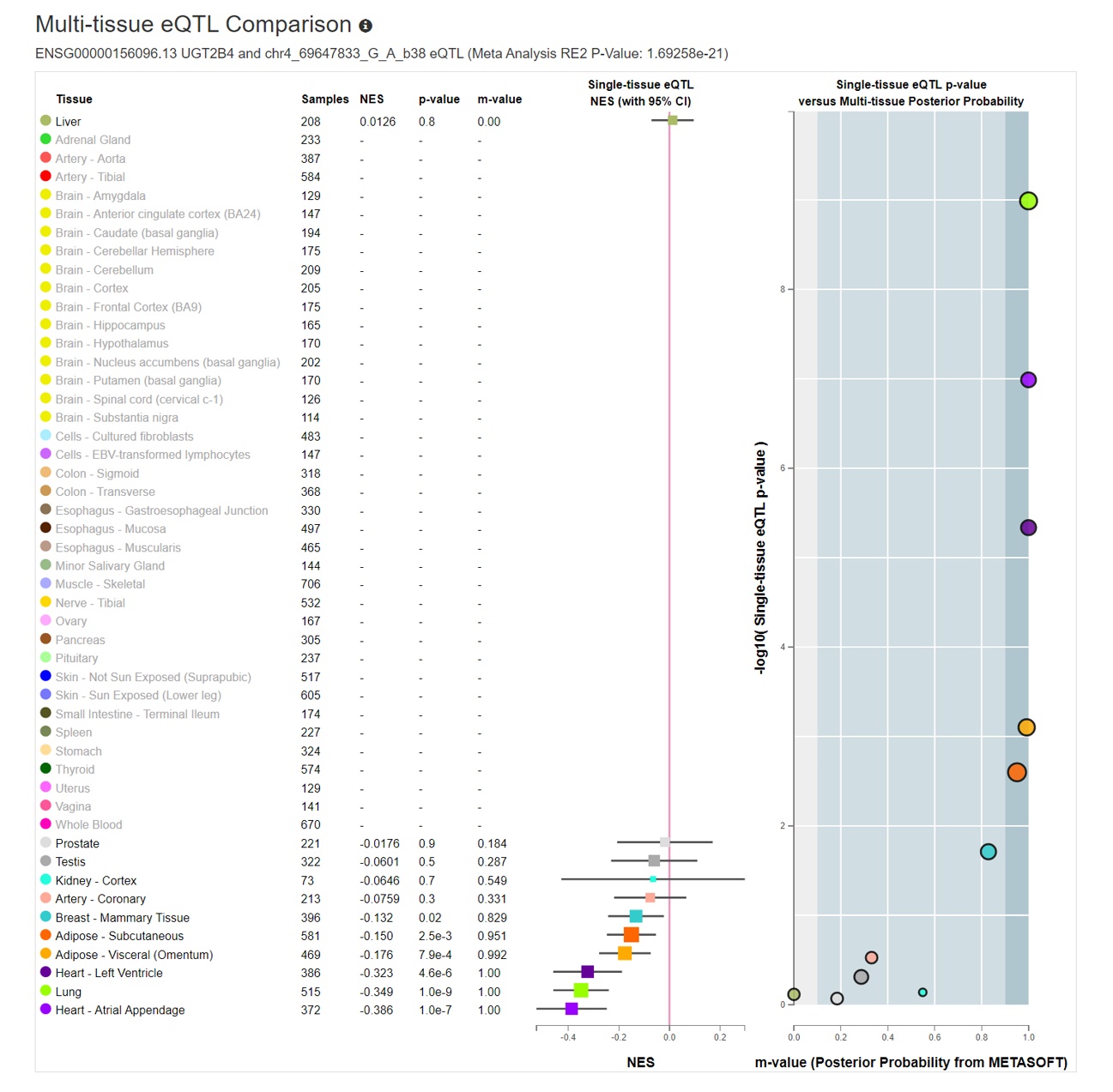
**
