# Supplementary figures and images for: A genome-wide association study identifies a novel association between SDC3 and apparent treatment-resistant hypertension
Source: BMC Med. 2022 Nov 30;20:463. doi: 10.1186/s12916-022-02665-x (PMC9710180; doi:10.1186/s12916-022-02665-x)

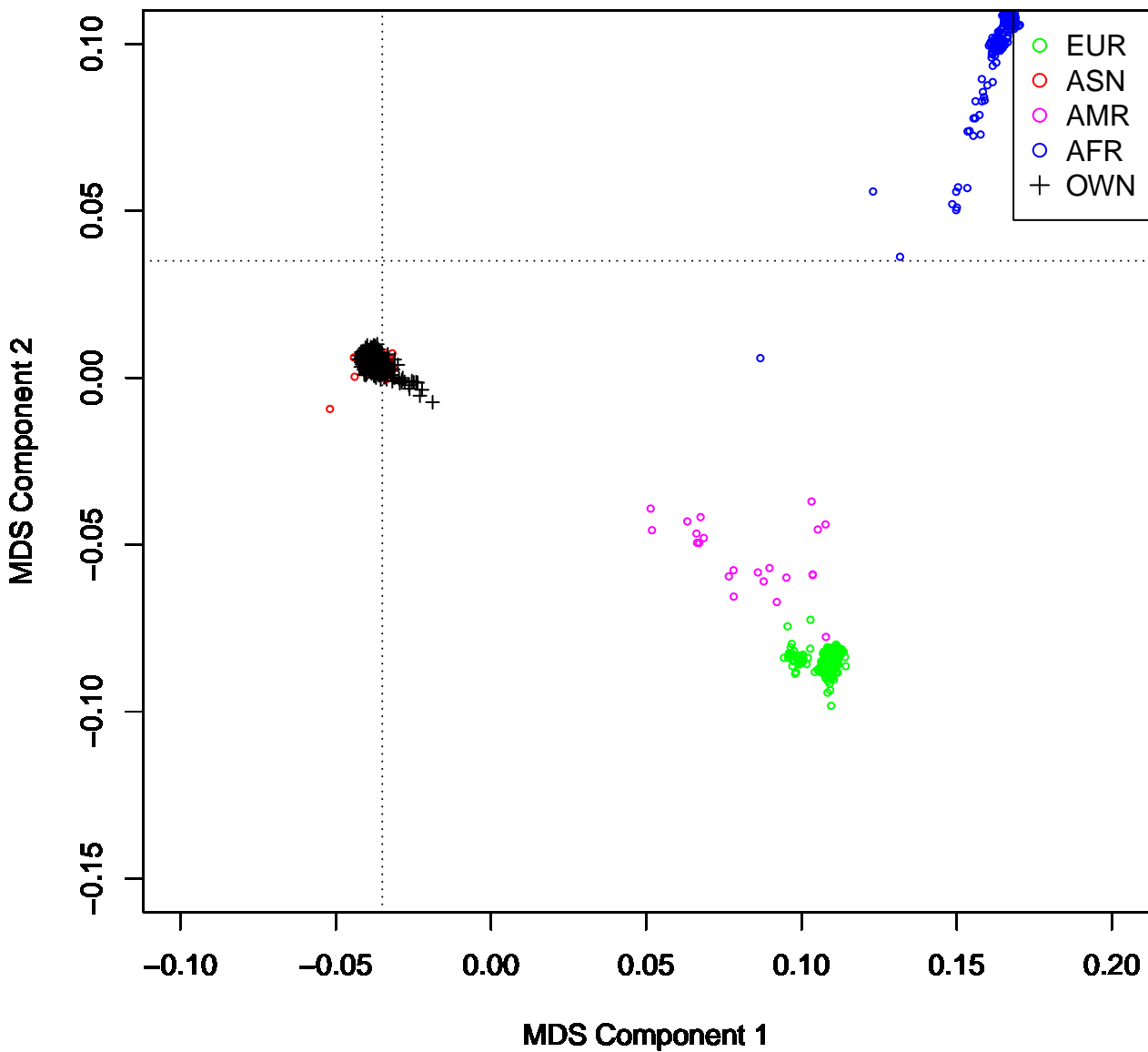

Supplement: Supplementary file 2 — Additional file 2. Multi-dimensional scaling (MDS) analysis of the genotypes. [file 12916_2022_2665_MOESM2_ESM.pdf]
